# Effect of Early-Stage Human Breast Carcinoma on Monocyte Programming

**DOI:** 10.3389/fonc.2021.800235

**Published:** 2022-02-14

**Authors:** Marina Patysheva, Irina Larionova, Marina Stakheyeva, Evgeniya Grigoryeva, Pavel Iamshchikov, Natalia Tarabanovskaya, Christel Weiss, Julia Kardashova, Anastasia Frolova, Militsa Rakina, Elizaveta Prostakishina, Lilia Zhuikova, Nadezhda Cherdyntseva, Julia Kzhyshkowska

**Affiliations:** ^1^ Laboratory of Translational Cellular and Molecular Biomedicine, Tomsk State University, Tomsk, Russia; ^2^ Laboratory of Tumor Progression Biology, Cancer Research Institute, Tomsk National Research Medical Center, Russian Academy of Sciences, Tomsk, Russia; ^3^ Laboratory of Genetic Technologies, Siberian State Medical University, Tomsk, Russia; ^4^ Laboratory of Molecular Oncology and Immunology, Cancer Research Institute, Tomsk National Research Medical Center, Russian Academy of Sciences, Tomsk, Russia; ^5^ Breast Cancer Unit, Cancer Research Institute, Tomsk National Research Medical Center, Russian Academy of Sciences, Tomsk, Russia; ^6^ Department of Medical Statistics and Biomathematics, Medical Faculty Mannheim, University of Heidelberg, Mannheim, Germany; ^7^ Tomsk Regional Oncology Center, Tomsk, Russia; ^8^ Institute of Transfusion Medicine and Immunology, Institute for Innate Immunoscience (MI3), Medical Faculty Mannheim, University of Heidelberg, Mannheim, Germany; ^9^ German Red Cross Blood Service Baden-Württemberg – Hessen, Mannheim, Germany

**Keywords:** monocytes, HLA-DR, CD163, RNA-seq, breast cancer

## Abstract

Circulating monocytes are a major source of tumor-associated macrophages (TAMs). TAMs in human breast cancer (BC) support primary tumor growth and metastasis. Neoadjuvant chemotherapy (NAC) is a commonly used treatment for BC patients. The absence of the response to NAC has major negative consequences for the patient: increase of tumor mass, delayed surgery, and unnecessary toxicity. We aimed to identify the effect of BC on the subpopulation content and transcriptome of circulating monocytes. We examined how monocyte phenotypes correlate with the response to NAC. The percentage of CD14-, CD16-, CD163-, and HLA-DR-expressing monocytes was quantified by flow cytometry for patients with T1-4N0-3M0 before NAC. The clinical efficacy of NAC was assessed by RECIST criteria of RECIST 1.1 and by the pathological complete response (pCR). The percentage of CD14+ and СD16+ monocytes did not differ between healthy women and BC patients and did not differ between NAC responders and non-responders. The percentage of CD163-expressing CD14^low^CD16+ and CD14+CD16+ monocytes was increased in BC patients compared to healthy women (99.08% vs. 60.00%, p = 0.039, and 98.08% vs. 86.96%, p = 0.046, respectively). Quantitative immunohistology and confocal microscopy demonstrated that increased levels of CD163+ monocytes are recruited in the tumor after NAC. The percentage of CD14^low^CD16+ in the total monocyte population positively correlated with the response to NAC assessed by pCR: 8.3% patients with pCR versus 2.5% without pCR (p = 0.018). Search for the specific monocyte surface markers correlating with NAC response evaluated by RECIST 1.1 revealed that patients with no response to NAC had a significantly lower amount of CD14^low^CD16+HLA-DR+ cells compared to the patients with clinical response to NAC (55.12% vs. 84.62%, p = 0.005). NGS identified significant changes in the whole transcriptome of monocytes of BC patients. Regulators of inflammation and monocyte migration were upregulated, and genes responsible for the chromatin remodeling were suppressed in monocyte BC patients. In summary, our study demonstrated that presence of BC before distant metastasis is detectable, significantly effects on both monocyte phenotype and transcriptome. The most striking surface markers were CD163 for the presence of BC, and HLA-DR (CD14^low^CD16+HLA-DR+) for the response to NAC.

## Introduction

Breast cancer (BC) is the most common cancer among women and the second most common overall (1). State-of-the art breast cancer treatment is a multimodal approach integrating surgery, radiation, and systemic treatment, where surgery is the most effective BC treatment. Neoadjuvant chemotherapy (NAC) is commonly used as therapy for breast cancer patients, who receive chemotherapy before surgery to reduce tumor size to preserve healthy breast tissue. Efficient response to NAC correlates well with more prolonged overall survival (2, 3). However, the absence of the response to NAC has significant negative consequences for the patient: increase in tumor mass, delayed surgery, and unnecessary intoxication.

The innate immune system controls primary tumor development, growth, angiogenesis, and metastatic spread (4). Innate immune systems, especially tumor-associated macrophages (TAMs), can both cooperate with chemotherapy and block its effects (5). Circulating monocytes are precursors for the majority of TAMs that control tumor growth and metastasis (5–8). Potentially, circulating monocytes can differentiate to tumor-killing macrophages. However, intratumoral microenvironments, including hypoxia, cancer cell-produced cytokines, and growth factors, promote both the recruitment of monocytes into tumor tissue and their differentiation toward tumor-supporting M2-like macrophages (9, 10). Tumor-associated macrophages (TAM) are the most common and functionally active innate immune cells in the tumor microenvironment (6–9). There is a high correlation of proliferating TAMs with low patient survival due to the high malignancy of the tumor (4, 11, 12). The functions of TAMs are controlled on the transcriptional, epigenetic, and metabolic levels (4). The differentiation of monocytes after their migration into tissues affects the TAM function and significantly affects intramural immune status, level of angiogenesis and lymphangiogenesis, proliferation of cancer cells, and efficiency of adaptive immune response (5, 13–15). In majority of cancers, including breast, lung, prostate, and ovarian cancer, TAM substantially support tumor progression (12).

The total increase in circulating monocytes correlates with a poor clinical outcome in oral, breast, gastric, and rectal cancer (16–19). Also, in breast cancer, a high level of monocyte chemoattractant protein 1 (MCP-1, CCL2) in the tumor tissue and in the circulating blood correlates with a poor prognosis (9, 20–22). Different subsets of monocytes can act as precursors of tumor-associated macrophages (TAMs), which have pro-tumor activity and are involved in stimulating the secretion of mediators by the tumor and recruiting other blood monocytes into the tumor tissue with their subsequent differentiation into TAMs (10–13). Systemic regulation of monocytes is possible through blood cytokines and chemokines, mediators of inflammation, exosomes, and lipid and carbohydrate metabolites produced by tumor (15, 23). Therefore, tumor has a potential to affect the content and phenotype of circulating monocyte subtypes before monocytes are recruited into tumor mass. Since the population of blood monocytes is heterogeneous, different subpopulations can react to the tumor presence and correlate with tumor characteristics and treatment efficacy (24–26). Despite extensively accumulating knowledge about the mechanism by which TAM decrease the efficiency of chemotherapy, information about the role of monocytes as regulators of tumor response to chemotherapeutic agents is extremely limited (27).

In our study, we checked the hypothesis that content and activation of circulating monocytes can be affected by the presence of a breast carcinoma, and monocytes can have determinants that predict tumor sensitivity to chemotherapy. We provide the evidence that the monocyte subpopulation marked by CD163 and the whole transcriptome of circulating monocytes is affected by the presence of tumor. We found that HLA-DR+ minor monocyte subsets are indicative for the chemotherapy outcome.

## Materials and Methods

### Patients

The study population of the discovery cohort consisted of breast cancer patients who were treated in the Cancer Research Institute, Tomsk National Research Medical Centre (Tomsk, Russia), from 2014 to 2021. All patients had an invasive breast carcinoma of no special type. The flow cytometry study cohort included 38 patients (**Table S1**). Patients received 4–8 courses of neoadjuvant chemotherapy (NAC) in accordance with the primary breast cancer: “ESMO Clinical Practice Guidelines for diagnosis, treatment, and follow-up 2015” (28) (**Table S1**). All patients were undergoing surgical treatment, radiotherapy, and an adjuvant chemotherapy after NAC. The RNA sequencing study included patients with breast cancer (n = 9) and healthy females (n = 7) (**Table S2**). Real-time PCR analysis enrolled independent from RNA sequencing a research cohort of 20 patients with breast cancer and 15 healthy females (**Table S2**). An immunohistochemistry (IHC) analysis included an independent group of 122 female patients with invasive breast carcinoma (**Table S3**). For the IHC analysis, patients were divided into two groups according to the neoadjuvant treatment: 1) patients who did not receive NAC (N = 26) and 2) patients who underwent NAC (N = 96). Patients with NAC received 6–8 courses of chemotherapy in accordance with the recommendation described above (28). Chemotherapeutic regimens included FAX (fluorouracil, adriamycin, and capecitabine), CAX (cyclophosphamide, adriamycin, and capecitabine), CMX (cyclophosphamide, methotrexate, and fluorouracil), CP (cisplatin plus cyclophosphamide), CAP (cyclophosphamide, adriamycin, and platinum), and taxotere.

All patients were assessed using the RECIST 1.1 criteria after all courses of NAC based on the results of clinical examination, breast ultrasound, and/or mammography. Complete response (CR) (100% of tumor reduction), partial response (PR) (decreasing in tumor volume by more than 50%), stable disease (SD) (decreasing in volume by less than 50% or no more than 25% of increasing), and progression disease (PD) (increasing in tumor volume by more than 25%) were registered. According to the international recommendations, patients with complete and partial response composed the group with objective response, and patients with stabilization or progression compiled the group with the absence of response to NAC (29). Histological components of the “Residual Cancer Burden” were retrieved for calculating the score as described by Symmans (30). The RCB index enables the classification of residual disease into four categories: RCB-0 (complete pathologic response = pCR), RCB-I (minimal residual disease), RCB-II (moderate residual disease), and RCB-III (extensive residual disease). RCB has been calculated through the web-based calculator that is freely available on the internet (www.mdanderson.org/breastcancer_RCB).

Healthy female volunteers were enrolled in this study as a control group (17 for flow cytometry analysis, 5 for bulk RNA sequencing, and 15 for real-time qPCR). The inclusion criteria for the healthy women cohort were as follows: (a) age from 36 to 70 years, (b) no active medical conditions, (c) not taking immunomodulatory medication (over the counter or prescription) within 30 days of study, (d) willing and able to provide an informed consent, and (e) no current or past history of an oncology disease.

### Peripheral Blood Mononuclear Cell Isolation and Multicolor Flow Cytometry Analysis

Whole-blood samples were obtained from the 17 healthy volunteers and 38 patients before any treatment procedures. The peripheral blood mononuclear cells (PBMCs) were separated from whole blood by density gradient centrifugation using Lymphoset, Lymphozyte Separation Media (Biowest, France), density 1.077 g/ml. The PBMCs were washed and lysed using VersaLyse buffer (Beckman Coulter, USA). After red blood cell lysing, PBMCs were incubated with fluorescence-labeled antibody cocktail: CD45-APC-Cy7, CD14-FITC, CD16-APC, CD163-PE, HLA-DR-PE-Сy5 (**Table S4**), and 7-aminoactinomycin D (7-AAD, BD Biosciences) for dead cell discrimination. Cells were incubated for 15 min in the dark at room temperature and analyzed within 30 min. For each sample, a minimum of 200,000 events were collected. The compensation procedure was performed using VersaComp antibody capture beads (Beckman Coulter, USA). Sample acquisition was performed on a NovoCyte 3000 cytometer (ACEA Biosciences, USA) and following the gating strategy shown in **Figure S1**. Data analyses were performed with NovoExpress software (ACEA Biosciences, USA).

### Monocyte Isolation for RNA Sequencing and RT-PCR Validation

Peripheral blood mononuclear cells (PBMCs) were separated from whole blood by density gradient centrifugation using Lymphoset, Lymphozyte Separation Media (Biowest, France), density 1.077 g/ml. After that, monocytes from the PBMC fraction were obtained by FACS. Cells were resuspended in 150 μl of staining buffer (Cell Staining Buffer, Sony, Japan). Monocytes were defined as CD45+CD56-CD14+7-AAD- population. Conjugated monoclonal antibodies to CD45, CD56, CD14, and 7-AAD were added to the cell suspension (online **Table S4**). Samples were analyzed on a MoFlo XDP cell sorter (Beckman Coulter, USA). Sorting of monocytes was carried out in the Purify 1–2 mode, the sorting efficiency was 70%, and the purity of the target population was 96%–99% (**Figure S2**). Monocytes for real-time PCR analysis were isolated from peripheral blood by density gradients followed by positive magnetic selection using CD14+ MACS beads (no. 130-050-201, Miltenyi Biotec, Germany), resulting in 90%–98% monocyte purity as confirmed by flow cytometry.

### RNA Extraction

RNA extraction total RNA was extracted from the lysed FACS-purified samples using the RNeasy Plus Micro Kit (Qiagen, USA). The quality of RNA was assessed by TapeStation 4150 automated electrophoresis system (Agilent Technology, USA). The RNA integrity index (RIN) was 9.0–9.9. The quantity of RNA was assessed by a Qubit 4 fluorometer (Thermo Fisher Scientific, USA). The amount of obtained RNA was 0.4–2.8 ng/μl.

### Whole-Transcriptome RNA Sequencing

RNA libraries were prepared with NEXTflex Rapid Directional qRNA-Seq Kit using indexed barcodes NEXTflex-qRNA-8nt-Barcodes (NOVA-5198-02, Bioo Scientific, PerkinElmer Applied Genomics, USA) according to the manufacturer’s protocols. Ribosomal RNA depletion was performed with NEBNext^®^ rRNA Depletion Kit (Human/Mouse/Rat) (NEB #E7400, New England Biolabs Inc., USA).

Whole-transcriptome sequencing was performed on a total of 9 samples of monocytes isolated from breast cancer patients and 7 healthy volunteers. Prepared libraries were then pooled and sequenced on an Illumina NextSeq 500 instrument (Illumina, USA) with NextSeq 500/550 High-Output v2.5 Kit (75 cycles) (cat #20024906). Raw data quality control was performed using FastQC (FastQC, RRID : SCR_014583) and visualized by MultiQC (MultiQC, RRID : SCR_014982) (https://pubmed.ncbi.nlm.nih.gov/27312411/). Read alignment was performed using a STAR aligner (STAR, RRID : SCR_004463) with GRCh38 genome and GENCODE annotations (https://pubmed.ncbi.nlm.nih.gov/23104886/). The numbers of reads assigned to genomic features were calculated using QoRTs software (QoRTs, RRID : SCR_018665) (https://bmcbioinformatics.biomedcentral.com/articles/10.1186/s12859-015-0670-5). Subsequent analysis steps were performed using DESeq2 software (DESeq2, RRID : SCR_015687) (https://genomebiology.biomedcentral.com/articles/10.1186/s13059-014-0550-8). Differential expression data were visualized with pheatmap (pheatmap, RRID : SCR_016418), EnhancedVolcano (EnhancedVolcano, RRID : SCR_018931), ggplot2 (ggplot2, RRID : SCR_014601), and Phantasus software (https://genome.ifmo.ru/phantasus). Fgsea (fgsea, RRID : SCR_020938) (https://www.biorxiv.org/content/early/2016/06/20/060012) and clusterProfiler (clusterProfiler, RRID : SCR_016884) (https://www.sciencedirect.com/science/article/pii/S2666675821000667) were used for gene set enrichment analysis of biochemical and regulatory pathways using gene lists ranked by expression level and p-value. GSEA results were visualized using ggpubr (ggpubr, RRID : SCR_021139) and GOplot (https://pubmed.ncbi.nlm.nih.gov/25964631/).

### Quantitative Real-Time PCR

The gene expression was quantified by quantitative real-time PCR using the TaqMan technology and was normalized to the expression of housekeeping gene glyceraldehyde 3-phosphate dehydrogenase (GAPDH). Primers were designed using the Vector NTI Advance 11.5.4 program and NCBI base. Primer synthesis was carried out by the DNA-synthesis company (Moscow, Russia). The complete sequences of used primers are listed in online **Table S5**. qRT-PCR was performed using the AriaMx Real-Time PCR thermocycler (Agilent Technologies).

### Immunohistochemistry

Formalin-fixed paraffin-embedded (FFPE) tissue sections were obtained from breast cancer patients. The antigen unmasking was performed using the PT Link module (Dako, Denmark) in T/E buffer (pH 9.0). Immunohistochemical staining was performed using monoclonal rabbit anti-CD163 (1:500, ab182422, Abcam) and visualized using the Polymer-HRP detection system (ab236466, Abcam, USA). The staining results were acquired by a Carl Zeiss Axio Lab.A1 light microscope (Jenamed, Carl Zeiss, Germany) and assessed as the percentage of area occupied by positive stromal cells over the total intratumoral stromal area (according to Salgado et al.) (31). Cells outside of the tumor border and around DCIS and normal lobules, as well as in tumor zones with crush artifacts, necrosis, and regressive hyalinization, were excluded.

### Immunofluorescence and Confocal Microscopy

FFPE tissue sections were obtained from 10 breast cancer patients. The antigen unmasking was performed using the PT Link module (Dako, Denmark) in T/E buffer (pH 9.0). For immunofluorescence (IF) staining, tumor FFPE clinical samples were treated with xylol solution and blocked with 3% BSA in PBS for 45 min, incubated with a combination of primary antibodies for 1.5 h; washed; and incubated with a combination of appropriate secondary antibodies for 45 min. Anti-CD163 rabbit monoclonal antibody (1:500, ab182422, Abcam), anti-CD68 monoclonal mouse antibody (1:100, NBP2-44539, clone KP1, Novus Biologicals), and anti-CD14 polyclonal sheep antibody (1:50, #BAF383, R&D Systems) were used. A combination of secondary antibodies was applied: Cy3-conjugated anti-rabbit, Alexa Fluor 488-conjugated anti-mouse (all donkey, dianova, Germany, dilution 1:400) and donkey Alexa Fluor 647-conjugated anti-sheep antibody (1:500, #A-21448, Thermo Fisher Scientific, USA). Samples were mounted with Fluoroshield Mounting Medium with DAPI (ab104135 Abcam, USA) and analyzed by confocal microscopy. Confocal laser scanning microscopy was performed with a Carl Zeiss LSM 780 NLO laser scanning spectral confocal microscope (Carl Zeiss, Germany), equipped with a ×40 objective. Data were acquired and analyzed with Black Zen software (RRID : SCR_018163). All four-color images were acquired using a sequential scan mode.

### Statistical Analysis

Statistical analysis was performed using SAS software, release 9.4 (SAS Institute Inc., Cary, NC, USA). Variable distribution was presented as median [Q1–Q3]. In order to compare monocyte expressions between 2 groups, Wilcoxon 2-sample tests were used. Furthermore, simple and multiple logistic regression analyses were performed in order to investigate the binary outcome “health status”. For each logistic regression analysis, the AUC (area under the curve) was assessed as a measure of goodness of the corresponding statistical model. A test with a p-value < 0.05 was considered statistically significant.

## Results

### CD14 and CD16 Do Not Reflect Effect of Breast Carcinoma on Monocytes

The baseline characteristics of patients are presented in **Table S1**. All patients were divided into two groups depending on age (less 45 years old and more 45 years old). There are different stages of BC which were included in this study depending on the tumor size and locoregional metastasis status (**Table 1**). All patients did not have distant metastasis. The study cohort consisted of 38 BC patients with three different molecular subtypes: Luminal B (n = 17), Her2+ (n = 7), and triple-negative (n = 14). The clinical response was detected in 35 patients after NAC, where 64% of the group had an objective response (n = 24); 36% in this group had no clinical response for NAC (n = 11); and for three patients the NAC course was abrogated due to poor drug tolerance.

**Table 1:** Flow cytometry analysis of CD14+16-, CD14+16+, and CD14^low^16+ in healthy female and cancer patients’ group.

	CD14+16-, %Median (Q1–Q3)	CD14+16+, %Median (Q1–Q3)	CD14^low^16+, %Median (Q1–Q3)
**Healthy**	86.12 (83.64–91.25)	3.68 (2.72–4.8)	2.70 (1.54–11.64)
**Breast cancer**	92.78 (83.60–98.87)	2.56 (1.44–5.60)	5.61 (1.23–8.1)

First, circulating monocyte subpopulations of breast cancer patients and healthy women were analyzed by flow cytometry. Monocyte subsets were identified according to the CD14 and CD16 expression into classical subpopulation (CD14+16-), intermediate subpopulation (CD14+16+), and non-classical subpopulation (CD14^low^16+).

Breast cancer patients and healthy women had a similar distribution of CD14 and CD16 markers in monocyte subpopulations, indicating that these two monocyte surface markers do not reflect the effect of breast cancer on monocytes, and a deeper analysis of the subpopulations and whole transcriptome is needed (**Table 1**).

### Elevated Levels of CD163 on Non-Classical Monocyte Subpopulation Are Indicative for the Presence of Breast Cancer

**Figure 1:**
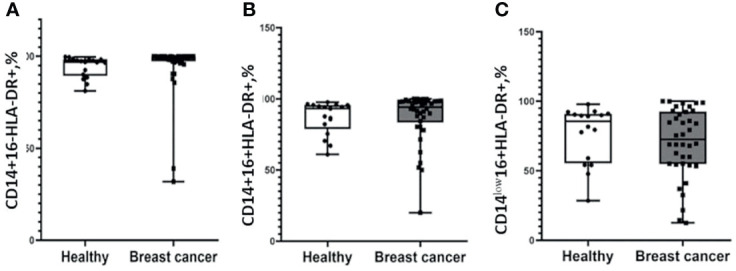
Patients with breast cancer and healthy female individuals have a similar distribution of HLA-DR-positive monocytes. Flow cytometry analysis of CD14+16-HLA-DR+ **(A)**, CD14+16+HLA-DR+ **(B)**, and CD14^low^16+HLA-DR+ **(C)**. Patients with breast cancer n = 38; healthy female individuals n = 17. Statistical analysis was performed by the Wilcoxon test.

Next, we analyzed the median proportion of cells with the expression of СD163 and HLA-DR on the CD14+16-, CD14+16+, and CD14^low^16+ subsets of monocytes in the study and control groups. Plots with gating strategies and expression histogram and gating strategy are demonstrated on the **Figure S1**. Analysis of the median proportion of HLA-DR+ classical, intermediate, and non-classical monocytes demonstrated similar parameters in the cancer group and healthy females (**Figure 1**).

**Figure 2:**
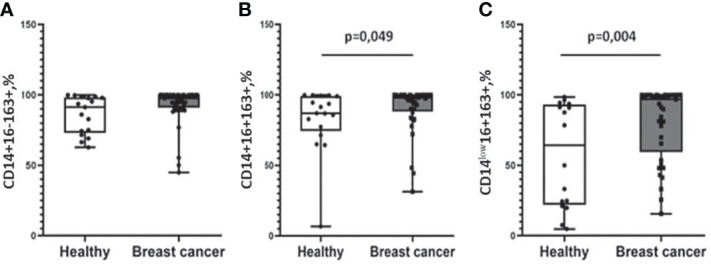
Differential expression of CD163 on monocyte subpopulations in patients with breast cancer patients and healthy female individuals. No differences in CD14+16-163+ monocyte subset distribution **(A)**. Patients with breast cancer were characterized by a significantly higher percentage of CD14+16+163 **(B)** and CD14^low^16+163+ **(C)** subpopulations compared with healthy women. Patients with breast cancer n = 38; healthy female individuals n = 17. Statistical analysis was performed by the Wilcoxon test.

We found higher median proportions of CD163-positive cells in the cancer group in CD14+16+ (98.08(86.40–100.00)%) and CD14^low^16+ (99.08(83.47–99.99)%) subsets compared to healthy women: 86.96(77.33–93.02)% for CD14+16+ (p = 0.049) and 60.00 (41.06–91.3)% for CD14^low^16+ (p = 0.004) cells (**Figure 2**). Moreover, using multiple logistic regression analysis with the binary outcome “health status,” we found that CD14^low^16+163+ monocytes were revealed to be the only significant variable for separating the two groups (odds ratio = 1.022, p-value = 0.015, AUC (area under the curve) = 0.745).

### Breast Cancer Alters Whole Transcriptome of Circulating Monocytes

**Figure 3:**
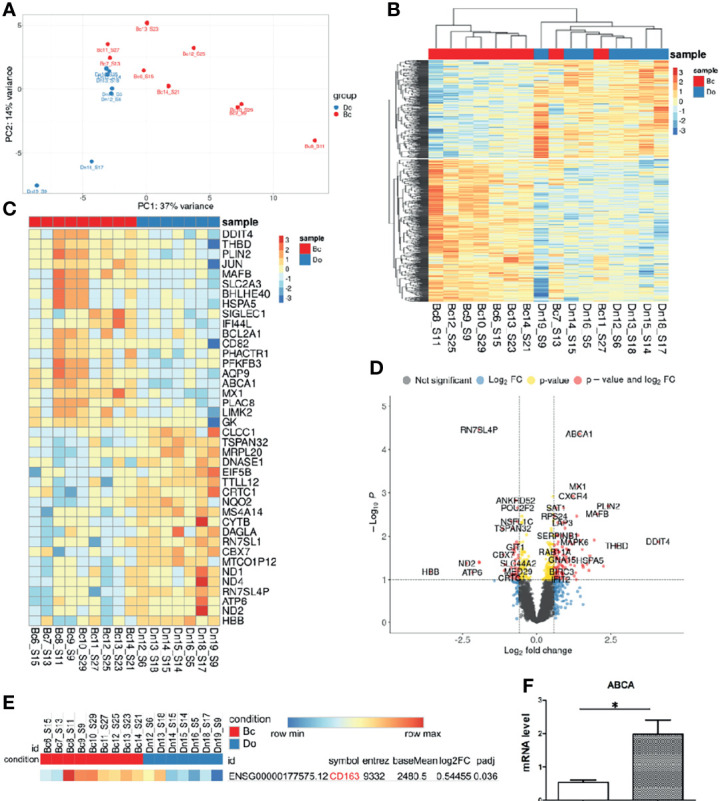
Breast cancer alters transcriptome of circulating monocytes. **(A)** Principal-component analysis (PCA) plot of genes expressed in monocytes from healthy female donors (Do), n = 7, and from breast cancer patients (Bc), n = 9. **(B)** Hierarchical clustering of all differentially expressed genes (DEGs) between BC and healthy monocytes. Expression values are *Z* score transformed. Samples were clustered using complete linkage and Euclidean distance. **(C)** Top 20 DEG log2FC genes in healthy individuals and breast cancer patients’ monocytes. **(D)** Volcano plot of RNA-Seq data breast cancer patients and healthy female monocytes. **(E)** *CD163* DEG in breast cancer and healthy female groups. **(F)** Expression of ABCA1 mRNA in breast cancer patient (n = 20) and healthy female (n = 15) monocytes (independent from the RNA-seq cohort), *p-value = 0.0006.

In order to examine the effect of the presence of breast carcinoma on the transcriptional programming of circulating monocytes, we compared the whole transcriptome of CD14+ monocytes from 9 patients with BC and 7 healthy female individuals by NGS (RNA-seq). On average, 14 million filtered and aligned reads were generated for each sample. Differential expression analysis (DEA) of monocytes from patients with breast cancer (BC) versus monocytes from healthy female individuals revealed 235 upregulated and 121 downregulated genes in BC monocytes (false discovery rate (FDR) < 0.1). Principal component analysis (PCA) and hierarchical clustering separated the transcriptome of BC monocytes from the transcriptome of healthy monocytes **Figure 3A**. Although there are outliers, principal component analysis (PCA) and hierarchical clustering segregated the transcriptomic profiles of normal monocytes and monocytes from breast cancer patients differently (**Figures 3A, B** **)**. The top significant genes are demonstrated by heatmap **Figure 3C**. A volcano plot shows genes (Log2FC > 0.58, FDR < 0.1) whose expression was significantly deregulated in breast cancer monocytes (**Figure 3D**).

The gene expression of *CD163* was upregulated in the breast cancer group with lg2FC = 0.54 and p-adj = 0.036 (**Figure 3E**) and correlated with flow cytometry analysis result (**Figure 2**). The top 20 upregulated genes such as *DDIT4*, *THBD*, *PLIN2*, *JUN*, *MAFB*, *SIGLEC1 ABCA1*, C*XCR4*, and *MX1* and other and the top 20 downregulated genes for monocytes from breast cancer patients, log2FC ≥ 0.58, FDR ≤ 0.05, were found (**Figures 3C, D** **)**. However, CD163 expression is not at the top 20 in BC monocytes, which can be explained by the elevation of CD163 only on the minor CD14^low^16+ monocyte subset, and for sequencing, we used the total pool of CD14+ monocytes (**Figure S2**). Validation of NGS data by qRT-PCR on monocytes isolated out of patients in the independent breast cancer cohort confirmed a significantly increased expression of the ABCA1 gene (**Figure 3F**). GSEA analysis reported enriched GSEA terms, such as an upregulated inflammatory response and migration in BC monocytes. Interestingly, downregulated were chromatin-remodeling pathways (**Figure S3** **)**. The GOChord plot showed pathway enrichment of selected DEGs in BC monocytes such as inflammatory pathways (inflammatory, INFy, INFa, and INFb responses) and hypoxia pathway (**Figure S4** **)**.

### CD163+ Monocyte-Derived Macrophages Are Accumulated in Breast Cancer Tissue Before and After Chemotherapy

**Figure 4:**
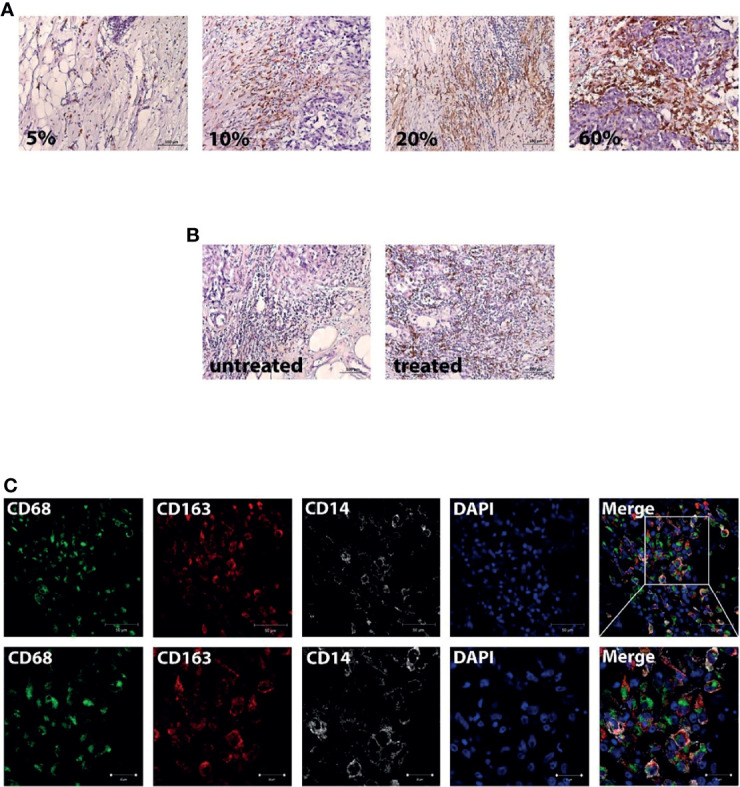
Breast cancer tissue is infiltrated by CD163-positive monocyte. **(A)** Examples of the percent content of the stromal surface area which was occupied by CD163+ cells. Scale bars correspond to 100 µm (×200). **(B)** Representative images from untreated and NAC-treated breast tumor tissue. Scale bars correspond to 100 µm (×200). **(C)** IF/confocal microscopy analysis was performed for breast tumor tissues. The infiltration of CD14+CD68+CD163+ cells was found in all samples. Representative images are demonstrated. Scale bar corresponds to 50 µm in the main image and 20 µm in the zoom image.

We compared the expression level of CD163+ macrophages in tumor tissue of patients without NAC and those who received NAC. The expression was assessed semiquantitavely similar to the recommendation of Salgado et al. (31). Stromal TAMs were scored as a percentage of the stromal areas alone excluding carcinoma cells. Examples of percent of area filled by CD163+ cells are presented in **Figure 4**. There, a score of 60% stromal cells means that 60% of the stromal surface area is occupied by CD163+ cells. We found that the percentage of area with CD163+ cells was higher in NAC-treated patients compared to untreated ones (10.0(5.0–20.0)%, mean = 14.06, N = 96 vs. 1.0(1.0–10.0)%, mean = 8.92, N = 26, p = 0.014) (**Figure 4**).

Then, we questioned whether NAC affects the accumulation of CD163-positive monocytes into breast cancer tissue. We performed IF/confocal microscopy analysis in tumor tissues taken after NAC. It was demonstrated that CD163 is predominantly expressed on CD14+CD68+ monocyte-derived macrophages, which infiltrate tumor mass (**Figure 4**), indicating that NAC can induce the recruitment of CD163+ monocytes into breast cancer tumor. The next question was to identify whether an additional marker on circulating monocytes can be indicative for the NAC efficiency.

### CD14^low^16+ and HLA-DR+ Monocytes and Response to Neoadjuvant Chemotherapy

We addressed the question, whether monocyte subtypes before NAC can correlate with clinical response to NAC. The differences between NAC non-responders and responders at CD14+16+ (1.33(0.52–3.10) vs. 2.24(1.34–5.31), p = 0.082) and CD14^low^16+ (5.45(2.01–10.23) vs. 2.24(1.17–4.67), p = 0.099) subsets before NAC slightly failed to reach statistical significance (**Table 2**). We found non-changed CD163+ cell proportions before treatment in the group with an objective response to NAC and the group without response to NAC in the CD14+16-, CD14+16+, and CD14^low^16+ subsets (**Table 2**).

**Table 2:** Flow cytometry analysis of CD14, CD16, and CD163 markers on monocytes from BC patients depending on clinical response to NAC.

Subset	BC without response, %, Median (Q1–Q3)	BC with response,%, Median (Q1–Q3)	Wilcoxon test, p-value
**CD14+16-**	92.4 (88.07–98.00)	93.14 (87.67–95.64)	0.986
**CD14+16+**	1.33 (0.52–3.10)	2.24 (1.34–5.31)	0.082
**CD14^low^16+**	5.45 (2.01–10.23)	2.24 (1.17–4.67)	0.099
**CD14+16-163+**	94.63 (90.74–97.49)	96.66 (90.05–99.86)	0.510
**CD14+16+163+**	96.30 (89.79–99.18)	98.78 (83.29–100)	0.590
**CD14^low^16+163+**	84.28 (33.11–99.02)	98.58 (61.64–99.99)	0.112

**Figure 5:**
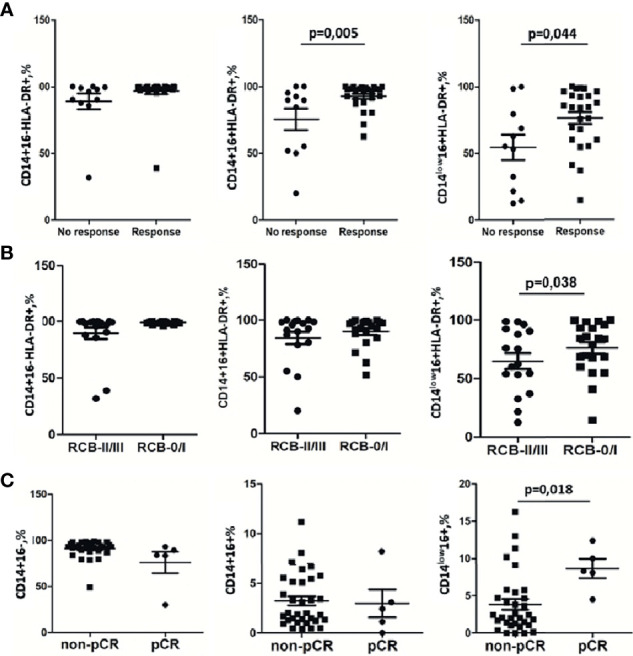
Monocyte subpopulations before treatment are sensitive indicators for NAC response. **(A)** Strong significant difference between the group with clinical response to NAC and the group without response to NAC detected by the RECIST 1.1 scale was found for CD14+16+HLA-DR+ and CD14^low^16+HLA-DR+. **(B)** CD14^low^16+HLA-DR+ subset decrease in the group of patients with RCB II/III vs. RCB 0/I. **(C)** CD14^low^16+ monocytes correlate with pCR. Statistical analysis was performed by the Wilcoxon test.

Before NAC, for HLA-DR we identified a higher proportion median of HLA-DR+ cells in the CD14+16+ subset: (97.72(91.28–98.87)%, p = 0.005) and in the CD14^low^16+ subset (84.62(63.98–93.16)%, p = 0.0447) for responders (**Figure 5A**). Accordingly, the non-responders’ group had a lower level of CD14+16+HLA-DR+ (84.51(51.77–92.59)%) and CD14^low^16+HLA-DR+ (55.12(21.70–79.32)%) (**Figure 5A**). The tendency for the increased expression of HLA-DR on CD14+16- monocytes was detected for NAC responders compared to non-responders. The percentage of CD14+16-HLA-DR+ out of all CD14+16- was 98.75(98.1–99.01)%) the responders, and 91.86(74.62–95.67)% for non-responders (p = 0.077) (**Figure 5A**). A multivariable logistic regression analysis with the binary outcome “response” provided a statistical model including CD14+16-HLA-DR+ with odds ratio = 0.88 (p = 0.019). Also, the statistical model includes CD14+16- (odds ratio = 2.1, p = 0.032) and CD14^low^16+ (odds ratio = 2.74, p = 0.019) either. The AUC of this model was 0.943.

The pathological complete response (pCR) is a clinically significant parameter for prediction of the long-term outcome in individual patients with early-stage breast cancer treated with preoperative systemic therapy (32, 33). We analyzed the correlation between pCR and monocyte subsets. The CD14^low^16+ subpopulation before NAC had a significant correlation with pCR (**Figure 5C**). Patients without pCR had 2.5 (1.3–5.45)% of CD14^low^16+ out of total monocytes, while patients with pCR had a significantly increased percentage of CD14^low^16+: 8.3(8.1–12.4)% (p = 0.018). The percentage of CD14+16- was similar in non-pCR 92.52 (88.07–94.58)% and pCR 83.78(83.48–89.29)% (**Figure 5C**). Similar data were obtained for the CD14+16+ subset: 2.03(1.39–5.59)% in the non-pCR group vs. 2.42 (1.13–3.10) in the pCR group (**Figure 5C**). Next, we evaluated the response to NAC by analysis of the tumor size in BC in patients using residual cancer burden (RCB) as a clinical parameter. Based on RCB grade, we have compared 2 patients’ groups, RCB-0/I group and RCB-II/III, and analyzed the percentage of CD14^low^16+HLA-DR+ monocytes in the CD14^low^16+ monocyte subpopulation. We found that in the RCB-0/I group CD14^low^16+HLA-DR+ constituted 84.28 (63.98–94.82)% and in the RCB-II/III group CD14^low^16+HLA-DR+ constituted 60.03 (32.5–82.73)% (p = 0.038; **Figure 5B**). These data corresponded to the data obtained for the monocytes subtypes’ correlation with NAC efficacy evaluated by the RECIST 1.1 scale (**Figure 5A**).

## Discussion

Systemic changes in the health status related to metabolic conditions and local processes characterized by inflammation result in change in the content of subpopulations and appearance of a non-typical biomarker on the circulating monocytes (23). In this study for the first time, we have identified the monocyte biomarkers indicative not only for the presence of breast cancer but also predicting the response of breast cancer patients to neoadjuvant chemotherapy, a broadly used approach to suppress the activity of primary tumor before the surgical intervention.

Chronic inflammation underlies the development of the most dangerous diseases, including malignant transformations (34–36). Monocytes can potentially sense the presence of tumor, and their clinical value was suggested (23). Up to date, the increased percentage of monocytes in the circulating mononuclear cells was found to be indicative for worse prognosis in cancer patients (16–19).

Isolated studies reported the correlations between main subsets of monocytes and clinical manifestation of cholangiocarcinoma (37), colorectal (38), and lung cancer (39). However, the data are still controversial, due to a lack of validation of the large samples, so there is no significant value for clinical use. Out study demonstrated that the main monocyte subsets (CD14+16-, CD14+16+, and CD14^low^16+) do not change in patients with breast cancer controlled by healthy individuals. Similar observations were made by other research groups who also did not find quantitative differences in the proportions of classical, intermediate, or non-classical subsets in breast cancer patients compared with healthy volunteers (40, 41). CD16 has been proposed to be a differentiation marker for monocytes, suggesting that CD14^low^16+ monocytes are more mature than CD14+16– monocytes (42). Therefore, breast cancer presence seems not to affect the monocyte differentiation or maturation in the circulation.

Searching for the informative biomarker for the systemic cross-talk between the growing tumor and the innate immune system, we found a high percentage of HLA-DR-positive cells within the CD14+16- subpopulation of monocytes. MHC class II surface protein HLA-DR is a key mediator of antigen presentation which is highly expressed in monocytes of healthy individuals. Only two patients had a decreased percentage of CD14+16-HLA-DR+ monocytes, but 38 women had similar data compared with the healthy group. The non-classical subset had a lower median of HLA-DR+ compared with the classical subset in the study and control groups. Interestingly, the CD14^low^16+HLA-DR+ percentage varied from 12.5% to 100% in BC patients and from 35.3% to 97.7% in healthy women. The statistical significance for the differences between breast cancer patients and healthy individuals was not achieved by analyzing the expression of HLA-DR on monocytes; however, we cannot exclude that statistical significance can be potentially achieved if larger patient cohorts are available. As we did not have a clear vision of the relevant effect sizes, we refrained from performing a statistical power analysis. Nevertheless, despite of the rather small sample sizes we obtained statistically significant results which may be clinically relevant. We suggest that studies with higher sample sizes should be performed in order to verify these results.

CD163 is a scavenger receptor for the hemoglobin–haptoglobin (Hb–Hp) complexes. In general, the cellular expression of CD163 is upregulated by anti-inflammatory factors, whereas pro-inflammatory signals downregulate its expression (43, 44). In healthy conditions, scavenging of Hb–Hp complex-mediated CD163 is silent and does not induce an inflammatory response in monocytes. Data regarding CD163 expression in the classical, intermediate, and non-classical monocytes are controversial. In colorectal cancer patients, CD163 expression was found to be decreased in the classical and total subpopulations (45). On the other hand, the CD163+14+ cell frequency in malignant pleural effusion was higher than that in non-malignant pleural effusion (46). BC patients demonstrated a higher level of CD14+163+ and CD14+CD163+CD204+ in a cohort of 56 women from Shanghai Sixth People’s Hospital (25). However, the authors did not analyze the distribution of CD163+ in classical, intermediate, or non-classical subsets. For the first time, we demonstrated that CD14+16+ and CD14^low^CD16+ (but not CD14+16-) had a significantly higher percentage of CD163+ positivity than the same monocyte subpopulations in healthy volunteers.

Considering that CD16 is indicative for the maturation of monocytes in circulation, we can hypothesize that CD163 expression is stimulated by the circulating factors produced by the tumor. According to multiple logistic regression analysis, the CD14+CD16++CD163+ subset was statistically significantly increased in patients with breast cancer. The role of CD163 as a marker of the M2 phenotype is highly questionable due to its expression of the macrophages in mixed chronic inflammatory conditions; however, CD163 is frequently used to identify tumor-supporting TAM in various types of cancer (12, 46–48). We proposed that CD163+ cells are a functional biomarker which does not strictly define the M2 direction of TAM (12). We found an increase in CD163 expression on overall monocyte pull by whole-transcriptome RNA sequencing. The skew of circulating monocytes to the scavenging direction in patients with breast, colorectal, and lung tumors indicate the appearance of the previously described tumor-educated monocytes (40, 49, 50).

In this study, we found the evidence that CD163 is elevated on the circulating monocytes in patients with breast cancer and is intensively recruited to the tumor site, suggesting that CD163 can be used as a marker for monocyte-derived TAMs. CD163+ TAMs are associated with poor histological grade, larger tumor size, Ki67 positivity, and LN metastasis in patients (51–53). A lot of studies from different cohorts of BC patients showed that CD163+ macrophages can be predictors of poor survival (54–58). Frequently, higher infiltration of TAMs expressing CD163 correlated with unfavorable clinic-pathological features and reduced survival in patients with breast cancer. Their polarization and localization in different tumor compartments should be taken into account for determining the prognostic and/or predictive role of TAMs. It was shown that CD163+ macrophages can have a positive effect depending on the local microenvironment in LN (58).

Predicting the response to standard NAC in advance, before the treatment start, is a highly beneficial strategy for the personalized optimization of cancer treatment and has a good potential to improve therapy outcomes and patient survival. Our results demonstrate a statistically significant correlation between the percentage of CD14+16+HLA-DR+ and CD14^low^16+HLA-DR+ and NAC efficacy. Patients who responded to NAC showed a higher level of HLA-DR+ monocytes in these subsets. Moreover, our statistical model included a CD14+16-HLA-DR+ variable with an odds ratio of less than one and a relatively high AUC value of 0.43. We suggest that an increased presence of CD14+16-HLA-DR+ is a good predictive marker because a higher percentage of this subset correlates with a small risk of non-response NAC.

In the last decade, CD14+HLA-DR^low^ monocytes were found in the blood of patients with B-cell lymphomas (59, 60) and glioblastoma (61), renal (62), and prostate (63) cancers. A low expression of HLA-DR on the CD14+ cells was associated with impaired immune function in many inflammatory diseases (64, 65). Therefore, a lower percentage of HLA+ monocytes correlates with immunosuppression.

## Conclusions

Monocytes are universal innate immune sensors for the non-self and unwanted-self circulating factors, including factors produced by a growing tumor. Based on our data, we can hypothesize that the systemically suppressed antigen-presenting ability of the innate immune system diminishes also the effect of NAC, and patients with a lower percentage of CD14+HLA-DR+ have a higher risk of unsuccessful response to NAC and should be subjected to radical surgery as soon as possible. However, this hypothesis needs further verification on the large patient cohorts. Overall, our study showed that human breast cancer on the stages before hematogenous, distant metastasis is detectable, growth tumor has a systemic effect on the innate immunity, and monocytes are circulating innate immune sensors for tumor presence.

## Data Availability Statement

The original contributions presented in the study are included in the article/**Supplementary Material**. Further inquiries can be directed to the corresponding author.

## Ethics Statement

The study was approved by the Local Committee for Medical Ethics of Cancer Research Institute of TNRMC (Russian Federation) and performed according to the guidelines of the Declaration of Helsinki and the International Conference on Harmonisation’s Good Clinical Practice Guidelines (ICH GCP) with written informed consent from all subjects. The patients/participants provided their written informed consent to participate in this study.

## Author Contributions

JKz, MP, MS, and NC desined the project. MP, IL, MS, EG, AF, EP, MR, JKa and NT performed the experiments. JKz, MP, PY, CW, and LZ analyzed the data. JKz, MP, NC, and CW wrote the manuscript. All authors contributed to the article and approved the submitted version.

## Funding

This research was funded by the Russian Foundation for Basic Research grant number 17-29-06037 and a state contract of the Ministry of Science and Higher Education of the Russian Federation “Genetic and epigenetic editing of tumor cells and microenvironment in order to block metastasis” no. 075-15-2021-1073.

## Conflict of Interest

The authors declare that the research was conducted in the absence of any commercial or financial relationships that could be construed as a potential conflict of interest.

## Publisher’s Note

All claims expressed in this article are solely those of the authors and do not necessarily represent those of their affiliated organizations, or those of the publisher, the editors and the reviewers. Any product that may be evaluated in this article, or claim that may be made by its manufacturer, is not guaranteed or endorsed by the publisher.
