# Supplementary material for: Effect of Early-Stage Human Breast Carcinoma on Monocyte Programming
Source: Front Oncol. 2022 Feb 14;11:800235. doi: 10.3389/fonc.2021.800235 (PMC8882686; doi:10.3389/fonc.2021.800235)
Supplement: Supplementary file 1 [file Image_1.pdf]

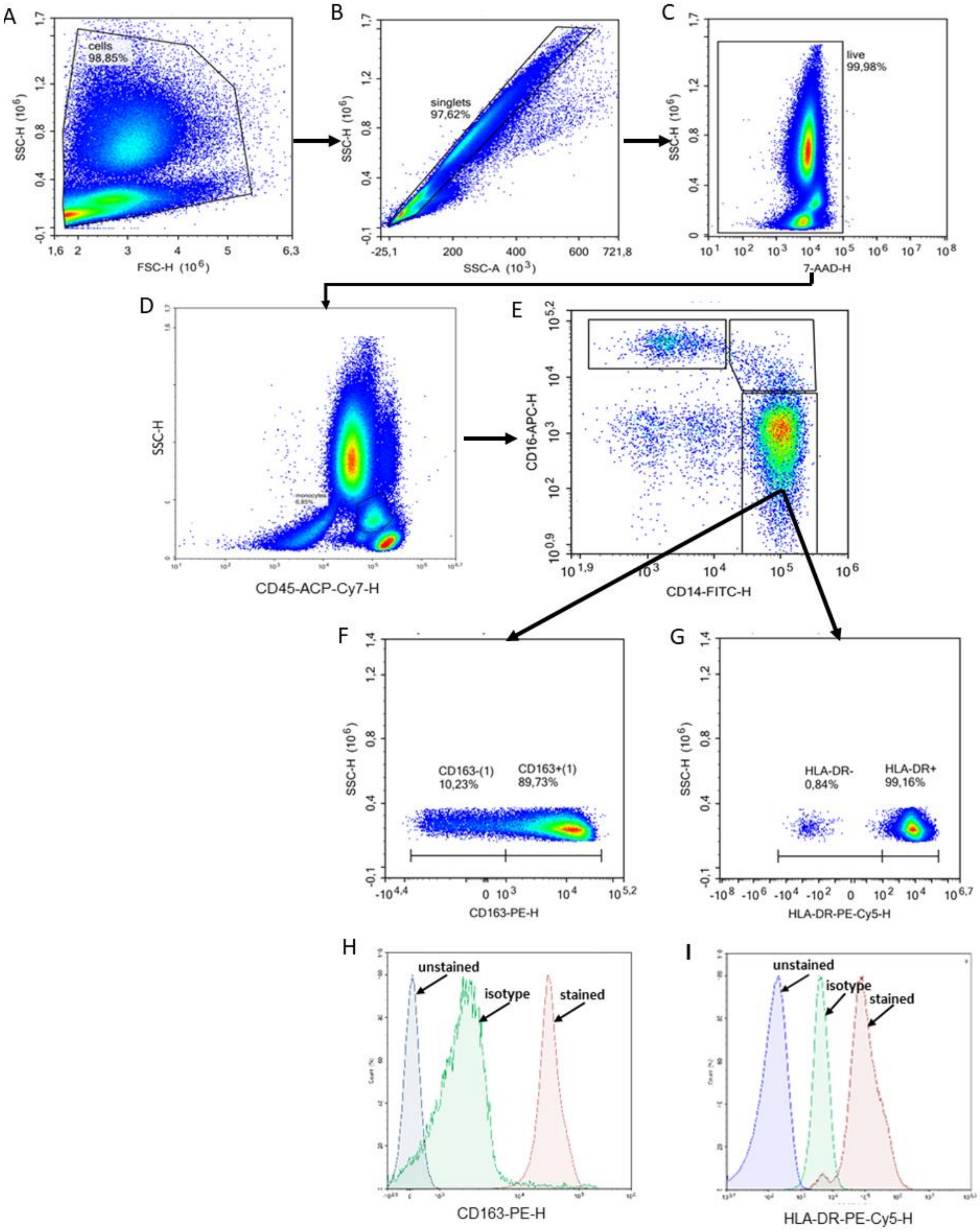

Figure S1. Flow cytometry gating strategy for the identification of human monocytes. A - broad selection of leucocytes based on their SSC/FSC properties; B - gating the cells that have an equal area and height, thus removing duplets (clumps); C - gating of live cells; D - monocytes among CD45 + cells; E - CD16 vs. CD14 plot: gating to select monocytes based on their characteristic "┐ " shape; F,G – CD163+ and HLA-DR+ selection from CD14+16- subset, selection from CD14+16+ and CD14^{low}16+ was carried out on the same principle; H,I – isotype control of CD163 and HLA-DR primary antibody conjugation.

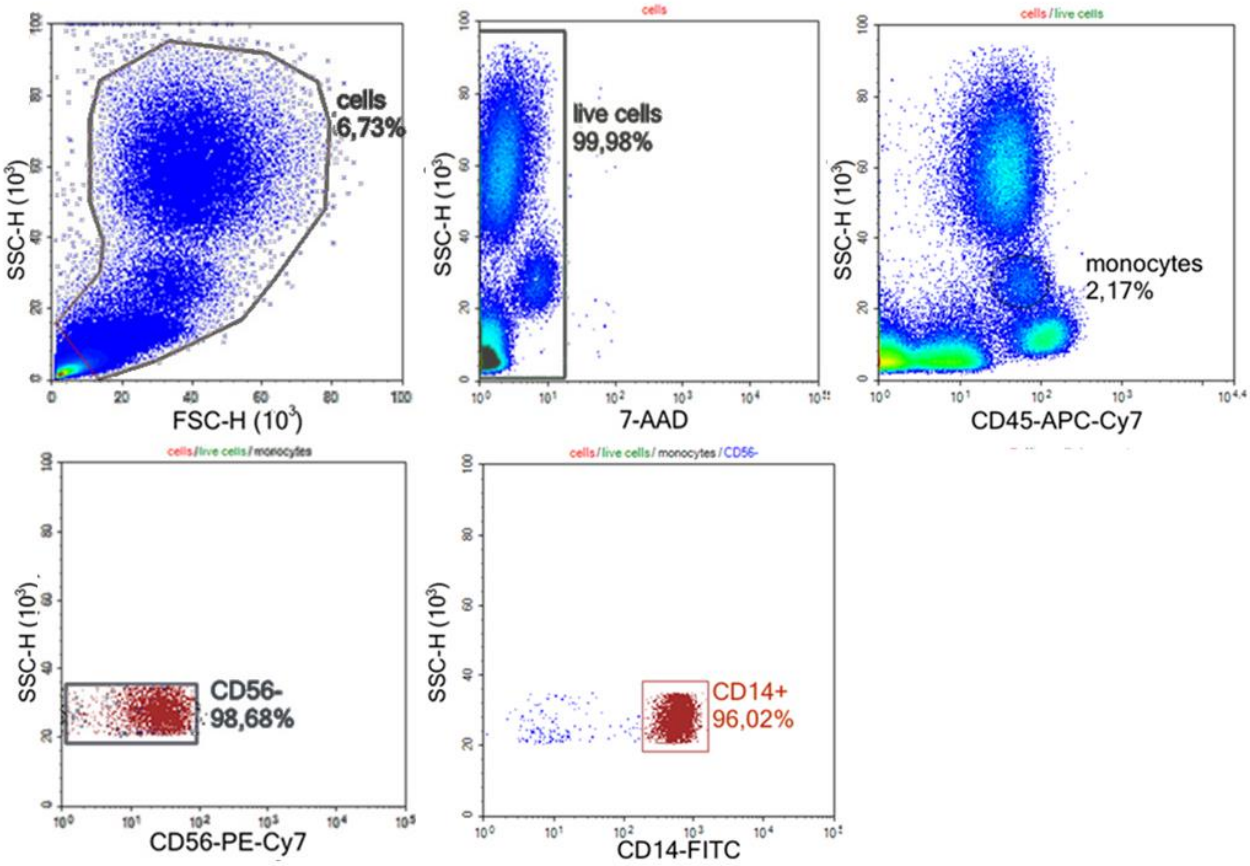

Figure S2. Flow cytometry gating strategy for the identification and isolation of human monocytes for transcriptomic analysis of the CD14+.

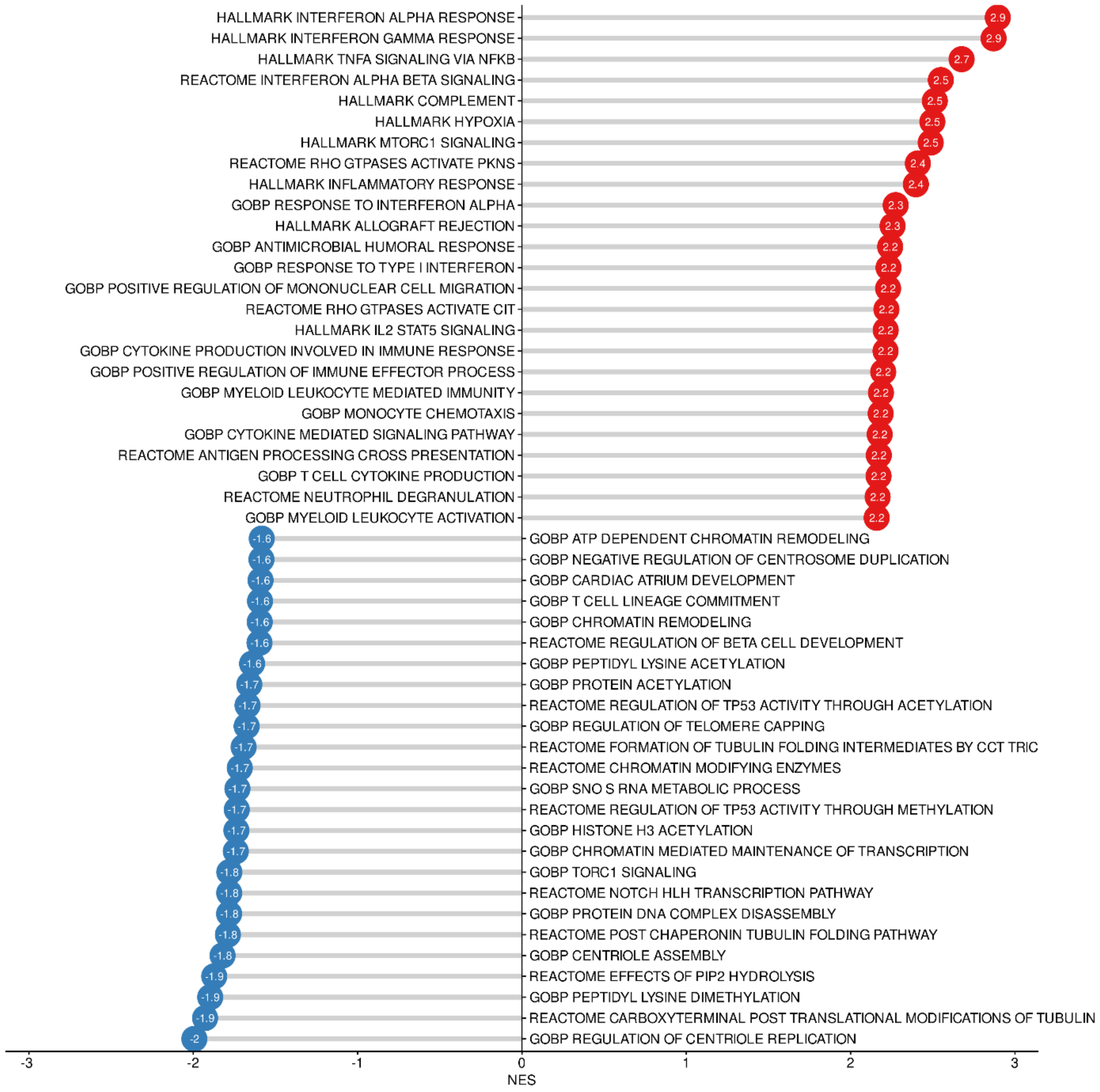

Figure S3. GSEA analysis of DEGs between cancer group and healthy group monocytes (blue, downregulated genes; red, upregulated genes)

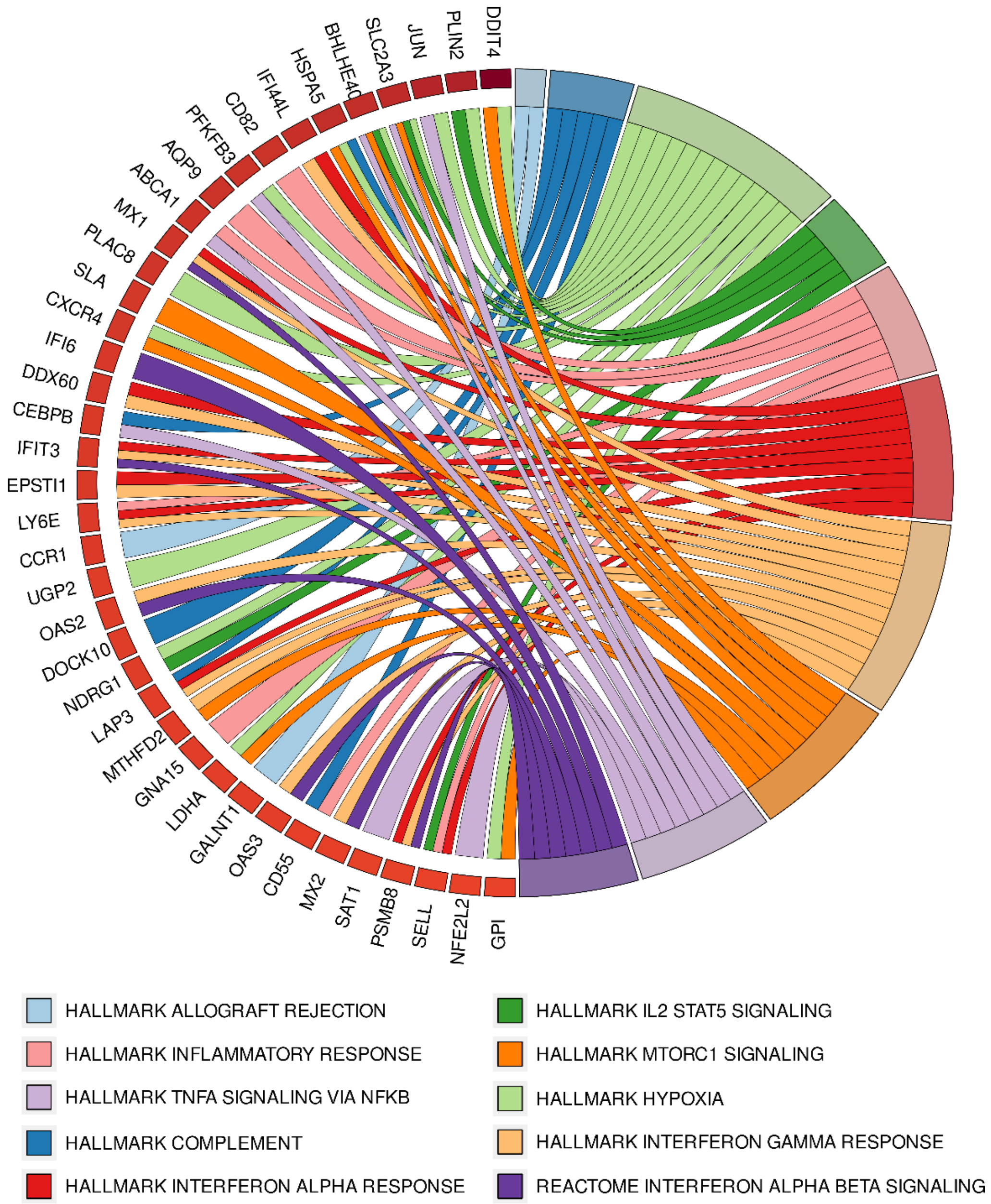

Figure S4. GOChord plot integrate genes expression data with the results of a functional pathways analysis.
