# Supplementary material for: Effect of Early-Stage Human Breast Carcinoma on Monocyte Programming
Source: Front Oncol. 2022 Feb 14;11:800235. doi: 10.3389/fonc.2021.800235 (PMC8882686; doi:10.3389/fonc.2021.800235)
Supplement: Supplementary file 2 [file Table_1.docx]

**Table S1. Summary of clinical characteristics of patients with breast cancer in flow cytometry study.**

|  | | **Number of Patients (N=38)** |
| --- | --- | --- |
| **Age (year)** |  | |
| ≤45  >45 | 25  13 | |
| **T stage** |  | |
| T1 | 4 | |
| T2 | 23 | |
| T3 | 2 | |
| T4 | 10 | |
| **N stage** |  | |
| N0 | 19 | |
| N1 | 12 | |
| N2 | 4 | |
| N3 | 3 | |
| **Molecular subtype** |  | |
| Luminal B | 17 | |
| HER2-positive | 7 | |
| Triple-negative | 14 | |
| **NAC scheme** |  | |
| AC+ Docetaxel or Taxotere | 18 | |
| АС or CP + Docetaxel or Taxotere + Pembrolizumab + Trastuzumab | 10 | |
| AC or CP | 7 | |
| **NAC clinical response** |  | |
| Complete response (CR) + partial response (PR) | 24 | |
| Stable disease (SD) + progression disease (PD) | 11 | |

|  | | **RNAseq**  **(N=9)** | **qPCR**  **(N=20)** |
| --- | --- | --- | --- |
| **Age (year)** |  | |  |
| ≤45  >45 | 4  5 | | 11  9 |
| **T stage** |  | |  |
| T1 | 1 | | 4 |
| T2 | 5 | | 11 |
| T3 | 1 | | 2 |
| T4 | 3 | | 3 |
| **N stage** |  | |  |
| N0 | 4 | | 7 |
| N1 | 4 | | 10 |
| N2 | 1 | | 2 |
| N3 | 0 | | 1 |
| **Molecular subtype** |  | |  |
| Luminal B | 5 | | 11 |
| HER2-positive | 1 | | 3 |
| Triple-negative | 3 | | 6 |

**Table S2. Summary of clinical characteristics of patients with breast cancer in bulk RNA sequence and real-time qPCR study.**

**Table S3. Clinical and pathological parameters of breast cancer patients enrolled in immunohistological analysis.**

|  | **Untreated patients (N=26)** | **Patients treated with NAC (N=96)** |
| --- | --- | --- |
| **Age**  ≤45  >45 | 19  8 | 69  27 |
| **Molecular subtypes**  Luminal A  Luminal B  Triple-negative  Her2-positive | 5  14  5  1 | 12  47  27  9 |
| **Lymph node status**  N0  N1-3 | 13  13 | 37  58 |
| **Hematogenous metastasis**  M0  M1 | 22  4 | 67  29 |

**Table S4.** **Antibodies and isotype controls.**

| **Antibodies** | **Clone** | **Isotype** | **Source** |
| --- | --- | --- | --- |
| CD45 - APC-Cy7 | 2D1 | Mouse IgG_1_, κ | BD Bioscience |
| CD14 - FITC | M5E2 | Mouse IgG2a, κ | BD Bioscience |
| CD16 - APC | 3G8 | Mouse IgG_1_, κ | BD Bioscience |
| CD163 - PE | GHI/61 | Mouse IgG_1_, κ | BD Bioscience |
| HLA-DR - PE-Сy5 | G46-6 | Mouse IgG2a, κ | BD Bioscience |
| CD56 – PE -Cy7 | NCAM16.2 | Mouse IgG1, κ | BD Bioscience |
| Isotype PE-Cy™5 | G155-178 | Mouse IgG2a, κ | BD Bioscience |
| Isotype PE | P3.6.2.8.1 | Mouse IgG1, κ | eBioscience, Thermo Fisher Scientific |

**Table S5. Sequences of primers with original design used for quantitative real-time**

**PCR analysis.**

| **Gene** | **Sequence** |
| --- | --- |
| CXCR4 | F 5'-GCAGCAGGTAGCAAAGTGA-3'  R 5'-CCTCGGTGTAGTTATCTGAAGTG-3'  Probe FAM 5'-CCTGAGTGCTCCAGTAGCCACC-3'BHQ1 |
| TCF7 | F 5'-GGCTTCTACTCCCTGACCT-3'  R 5'-GTGACCAGGTACACCAGAAC-3'  Probe FAM 5'-GTTCACCCACCCATCCTTGATGC-3'BHQ1 |
| DDIT4 | F 5'-GTGTCAGGGATCACTTGGGA-3'  R 5'-AGATGGAAGACCCAGATGGC-3'  Probe FAM 5'-AGTTGAGCTGGCAGGGGAGG-3'BHQ1 |
| PLIN | F 5'-TTGATCCACAACCGAGTGTG-3'  R 5'-CTAGCTTCTGGATGATGGGC-3'  Probe FAM 5'-TCATGTCCTCAGCCTATCTCAGTACAAA-3'BHQ1 |
| THBD | F 5'-AACAATGTGCTCTCGGGTTG-3'  R 5'-TGAAAATCAGAGATGGTGCC-3'  Probe FAM 5'-TGTTCACTTTTCCTCCCTCAGTGCC-3'BHQ1 |
| ABCA1 | F 5'-AAAACAGTTAATGACCAGCCACG-3'  R 5'-TCTGAAAGTGAGGTTCTTCC-3'  Probe FAM 5'-TGGCTGAGGGAACATGGCTTGTT-3'BHQ1 |
| GAPDH | F 5'-GCCAGCCGAGCCACATC-3'  R 5'-GGCAACAATATCCACTTTACCAGA-3'  Probe FAM 5'-CGCCCAATACGACCAAATCCG-3' BHQ1 |
